# Green exercise and mg-ca-SO_4_ thermal balneotherapy for the treatment of non-specific chronic low back pain: a randomized controlled clinical trial

**DOI:** 10.1186/s12891-019-2582-4

**Published:** 2019-05-17

**Authors:** Daniela Huber, Carina Grafetstätter, Johanna Proßegger, Christina Pichler, Ewald Wöll, Martin Fischer, Martin Dürl, Karin Geiersperger, Melanie Höcketstaller, Stefan Frischhut, Markus Ritter, Arnulf Hartl

**Affiliations:** 1 Institute of Ecomedicine, Paracelsus Medical University, 5020 Salzburg, Austria; 2 Department of Physiotherapy, Salzburg University of Applied Science, 5412 Puch/Urstein, Austria; 3 Departments of Internal Medicine and Orthopedics, General Public Hospital St. Vinzenz, 6511 Zams, Austria; 4 Ordination Dr. Stefan Frischhut MSc., Maximilianstr. 2, 6020 Innsbruck, Austria; 5 Institute of Physiology and Pathophysiology, Paracelsus Medical University, 5020 Salzburg, Austria; 6 Ludwig Boltzmann Institute for Arthritis and Rehabilitation, Department for Radon Therapy Research, Paracelsus Medical University, 5020 Salzburg, Austria

**Keywords:** Chronic non-specific low back pain, Balneotherapy, Spa therapy, Magnesium-calcium-sulfate thermal water, Mountain hiking, Green exercise, Moderate altitude, Nature therapy, Alpine environment, Mountain exercise

## Abstract

**Background:**

Non-specific chronic low back pain (nscLBP) has a high socio-economic relevance due to its high incidence, prevalence and associated costs. Therefore, it is essential to evaluate effective therapeutic strategies. This study examines the effects of moderate mountain exercise and spa therapy on orthopedic and psychophysiological parameters. Based on a three-armed randomized controlled trial, guided mountain hiking tours and balneotherapy in thermal water were compared to a control group.

**Methods:**

Eighty patients with diagnosed nscLBP were separated into three groups: The two intervention groups GE (green exercise) and GEBT (green exercise and balneotherapy) undertook daily mountain hiking tours, whereas the GEBT group got an additional treatment with baths in Mg-Ca-SO_4_ thermal water. The third group (CO) received no intervention. GE and GEBT group were treated for 6 days; all groups were followed up for 120 days.

**Results:**

Compared to GE and CO group, the GEBT treatment showed significant improvements of pain, some orthopedic parameters, health-related quality of life and mental well-being in patients with nscLBP.

**Conclusions:**

The results of this study confirmed a benefit of mountain hiking combined with Mg-Ca-SO_4_ spa therapy as a multimodal treatment of patients with nscLBP. Further studies should focus on long-term-effects of this therapeutic approach.

**Trial registration:**

ISRCTN, ISRCTN99926592. Registered 06. July 2018 - Retrospectively registered.

## Background

### Socioeconomic impact of low back pain

In industrialized western countries, complaints of lower back pain are one of the leading health problems regarding incidence and prevalence. This problem occurs in almost all population groups and is responsible for a considerable extent of medical and social services, and consequently also for high macroeconomic costs. An Austrian health survey conducted in 2015 showed that chronic back pain is the most frequent chronic illness in Austria: 1.8 million people were affected, which accounts for 24.4% of the population [1].

Furthermore, a characteristic sex- (women 25.8%, men 22.9%) and age-dependency was observed. In 2015 there were 37,463 acute hospital stays due to complaints in the back with an average length of stay of 6.9 days, with 139 persons under 15 years, 5907 persons aged 15 to 44 years, 11,984 persons aged 45 to 64 years and 19,433 persons aged 65 years and older [1]. The Austrian federal pension fund (Pensionsversicherungsanstalt, PVA) approved 55,807 orthopaedic inpatient rehabilitation proposals in 2016, of which 1548 were related to chronic back pain. In Austria, there is also the possibility to apply for cure vacations, which is a stay at a health resort with the main goal of maintaining the working ability, where the costs are partly covered by the insurance. In 2016, the PVA granted 87,640 orthopaedic cure applications, 5909 of them for chronic back pain. According to the PVA, a curative stay in 2016 costed in average about 1900 Euros and a stationary rehabilitation program approximately 3600 Euros, which in turn allows conclusions to the total costs [2]. Additionally to the direct costs of hospitalization and rehabilitation, low back pain also incurs considerable indirect costs due to incapacity, disability and early retirements. The drug treatment of pain patients causes further annual costs of more than 1.6 billion Euros and analgesics comprise one of the most frequently prescribed groups of medicines in Germany [3]. These data demonstrate the socio-economic relevance of this topic.

### Etiology and duration of low back pain

Discomfort of the lower back, located centrally and paravertebrally to the spine - caudal to costal arch and cranial to os coccygis -, with or without radiations, is called low back pain (LBP). According to causality, a distinction is made between specific LBP, describing pain of a diagnosable genesis, and non-specific LBP (nscLBP), in which no clear diagnostic indications of a specific cause, i.e. a central pathomechanism or an irritated structure, can be detected [4]. About 85% of the affected patients suffer from nscLBP [5].

In relation to the course of time, acute pain (less than 6 weeks), subacute (less than 12 weeks), and chronic or chronically recurrent (more than 12 weeks) back pain are differed [6]. Acute non-specific back pain is usually self-limiting, with a rate of convalescence of 90% within the first 6 weeks. Chronification of back pain can be assumed after 12 weeks (subacute phase) of therapeutic intervention without relief of symptoms, which is the case in two to 7 % of patients [5].

Using the hypothesis of the biopsychosocial pain model as an explanation for nscLBP, the complexity of this disease pattern becomes clear: The origin and duration of back pain is not only depending on physical (e.g. diminished muscle function, impairment of tissue repair), but also psychological (e.g. self-efficacy claims, solution competence) and social factors (e.g. work history, family expectations) [7]. However, in respect to the heterogeneous patient group, a precise etiology and pathogenesis of this kind of chronic musculoskeletal pain is still elusive [8].

### Therapy options of non-specific LBP

Due to multimodal causes and complex interactions of biological, psychological and social factors, nscLBP cannot be cured persistently, but various conservative therapy options to reduce pain and impairment are available, addressing the consequences of long-term pain [9]. Preference is given to conservative non-pharmacological treatments [9], with a wide range of recommendations. There are more than 50 different potential therapies promising pain relief or even healing, but only few have been thoroughly evaluated by evidence-based methods [10]. For patients already suffering from back pain, recurrences and chronification can be prevented best by multimodal programs, which means the combination of different sorts of therapy [11]. Two-thirds of systematic reviews covering this topic emphasize the need for new high-quality therapeutic studies [12].

Based on these recommendations, we investigated the following therapy concept for the treatment of patients with nscLBP: green exercise (active therapy combined with experience of nature) and balneotherapy.

Physical activity in natural environments such as meadow, forests or alpine pastures, is called green exercise. This nature therapy addresses patients in many ways [13–16]. Current evidence on green exercise refers to three main areas: regulation of immunological and physiological (stress) responses, improvement of psychological states and facilitation of health-promoting behavior [17]. Although, valid data elevating effects of green exercise in the treatment of nscLBP is still missing.

In addition to exercise, balneological treatment can be seen as a meaningful therapeutic option. Despite limited available data, there is encouraging evidence that balneo or spa therapy may be effective in the treatment of nscLBP [18]. As trials have reported, patients with nscLBP show improvements in pain, functionality and psychological parameters after balneological intervention [19]. However, further randomized controlled trials (RCTs) with high scientific quality are necessary to investigate this kind of therapy more accurately [20].

This RCT was conducted to find out whether green exercise - in this case moderate mountain hiking - combined with Mg-Ca-SO_4_ thermal balneotherapy is an effective and economic nature therapy to reduce symptoms (like pain, physical disability, quality of life, depression) and improve wellbeing of patients with nscLBP. Furthermore, we investigated whether green exercise or the combination of green exercise and spa therapy in Mg-Ca-SO_4_-type water discloses specific effects in intergroup comparisons. This RCT examines the hypothesis, whether a multimodal therapy (spa therapy and green exercise) can reduce the symptoms and improve the spinal mobility of patients suffering from nscLBP, in comparison to an intervention group without spa therapy and a non-intervention control group.

## Materials and methods

### Subjects

Eighty patients (35 men, 45 women, 19 to 65 years old) were included in this study. The participants were primarily recruited all over Austria through communication via the *Wasser Tirol* web page (www.albenbad.at), advertisements in newspapers and by physicians. Requirements for study inclusion were diagnosed nscLBP pain and repeated medical treatment because of nscLBP during the past 3 years. Therefore, a medical certificate from the respective treating doctor was mandatory for inclusion. Exclusion criteria were as follows: malignant diseases, previous operations in the lumbar spine area, suspected disc herniation, acute pain, confirmed osteoporosis, contraindications of balneotherapy (cardiovascular dysfunction, such as unstable hypertension, angina pectoris, thrombosis; pulmonary dysfunction; endocrine disorders like hyperthyroidism and hyperparathyroidism; other uncontrolled metabolic disorders like diabetes mellitus, active infectious diseases, incontinence etc.), hernia or pregnancy. No sample size calculation was possible with the available data at the time of study proposal for the selected outcomes.

The Ethics Committee of Salzburg (415-E/1487/4–2012) approved the study protocol and all participants gave written consent. This study adheres to CONSORT guidelines for reporting clinical trials.

### Study design/interventions

The study was set up as a randomized controlled clinical trial with three arms. Except for the non-intervention group (control/CO, *n* = 27), all participants spent 8 days at the village of Grins (Tyrol, Austria, 47°08′30.1″N 10°30′55.2″E), hosted in comparable hotels and receiving the same meals. The study procedure was carried out according to the following structure: Medical examinations were performed at arrival (day 0, T1), after the intervention (day 8, T2) and after 4 months (day 120, T3). The arrival of the patients took place on Saturday followed by medical measurements and an anamnesis of medical history at the same day. From Sunday to Friday all persons undertook daily guided hiking tours in the mountains. The structured movement program included a daily 5 h hiking tour in and around the municipality of Grins. The weekly hiking program is shown in Table 1.

**Table 1:** Intervention program

	Sea level starting point (= end point)	Sea level highest point	Elevation gain	Kilometers
Sunday	1459	1842	386	7.85
Monday	1003	1242	434	13.18
Tuesday	1000	1528	632	15.20
Wednesday	1002	1358	377	6.92
Thursday	989	1609	657	8.17
Friday	1025	2290	921	9.61
Total			3407	60.93

[sea level] = m a. s. l.

The first group (*n* = 27) only participated in green exercise/GE, whereas the second group (*n* = 26) got an additional treatment with baths in thermal water in the *Albenbad* (green exercise and thermal balneotherapy/GEBT). The baths in a tub lasted 20 min. Thereafter, the patients could relax in a heated (room temperature) resting tent. The *Albenbad* houses two bathtubs; after each patient, the water was omitted, the tub was carefully cleaned, disinfected and newly filled with warm (37 °C) thermal water.

On the following Saturday all patients took part in identical medical assessments as at the arrival day and departed after finishing them. This procedure was repeated five times with about ten patients per week.

Since blinding of modes of (non-)intervention has not been possible (specific smell of the thermal water), the inclusion of a control group (CO) without any intervention was necessary. The participants of this group received no intervention and stayed at home, maintaining their usual lifestyle. In return for their participation the CO group participants received an amount of 150 Euros per person to cover their study-prone expenses. The patients of the GE and GEBT group got a one-week free holiday including food and lodging in hotels in the village of Grins in Tyrol, Austria. Patients were free to discontinue participation in the study at any time without giving reasons.

**Fig. 1:**
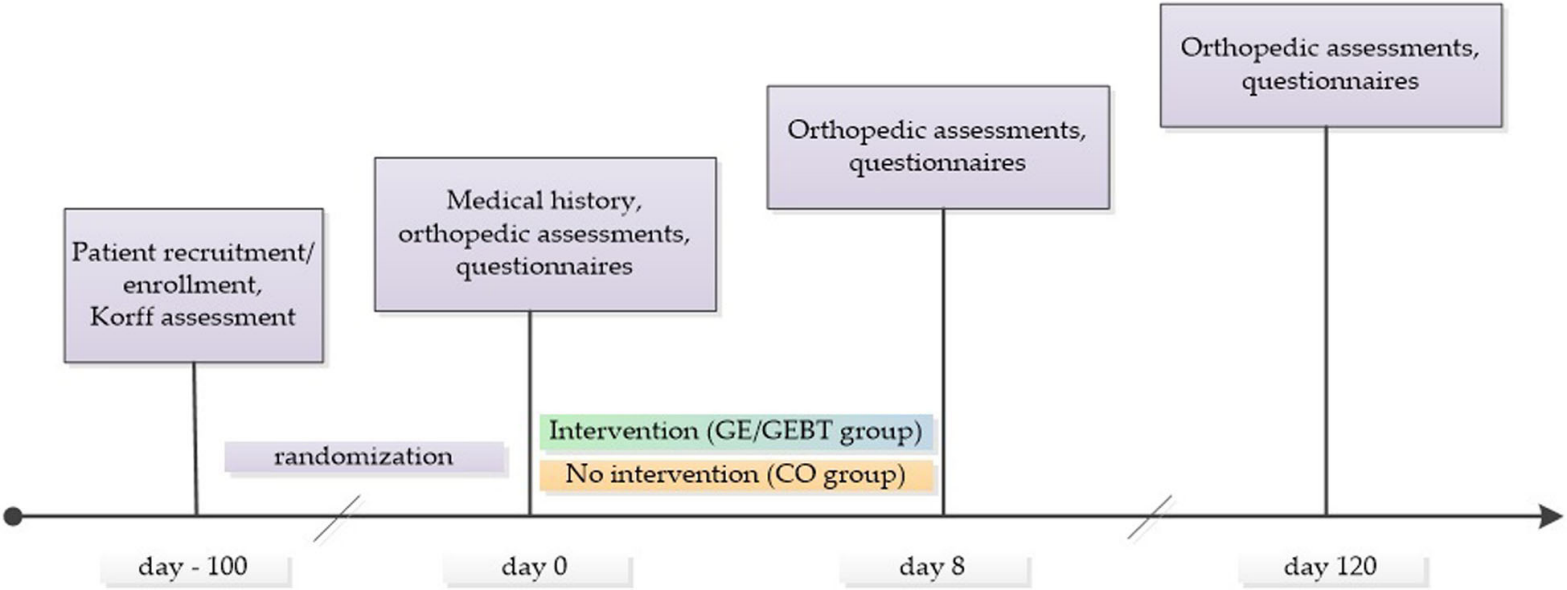
Time Schedule. Schematic chronological process of the controlled clinical trial

The study was executed from September 2013 to January 2014 for the first two groups (GE and GEBT) and from September 2014 to January 2015 for the third group (CO) to exclude seasonal effects. Due to personnel restrictions, a parallel assessment of all three study groups was not feasible. Orthopedic, physiological and psychological data from day 0 and 8 were collected on-site in a medical field laboratory in Grins. Follow-up examinations on day 120 of the intervention-group members as well as all examinations of the CO group members were conducted at the Paracelsus Medical University of Salzburg, Austria. The participants were stratified by the Korff assessment (pain related disability) [21] and were separated into the three groups by “Random Allocation Software 2.0” via block randomization. The schematic chronological sequence of this controlled clinical examination is shown in Fig. 1.

### Environmental parameters of the Albenbad water

This randomized controlled study was performed in Grins, which is located 1015 m above sea level in the district of Landeck (Tyrol, Austria) in the submontane of the Lechtal Alps. In 2007, this village obtained the official therapeutic and thermal water status for its water. The water produced by the three springs derives predominantly from a deep-seated aquifer with a subordinated admixture of young water, which duration of dwell time can be estimated to be less than 50 years. From age modeling the residence time of the old groundwater component was estimated to be between 10′000 and 30′000 years. The water of the wells is very well protected by the surrounding. It is of the rare Mg-Ca-SO_4_ – type with a 1.2 ratio of Ca/Mg. An overview of the water analysis can be seen in Table 4 in Appendix. This composition makes this water very interesting for medical application [22].

In 2010, a new bathing facility called *Albenbad* was established, in which the balneological part of the study was carried out. In the *Albenbad,* people can experience the water in form of undergoing Kneipp cures, drinking cures or bath cures. Additionally, a regional network of hiking trails, the *Albigen* paths, was set up in 2011, which also constituted the basic infrastructure for the present investigation.

### Assessments

At the beginning and end of the one-week intervention, as well as 4 months after the intervention, the functional spinal mobility was measured by parts of the Back Performance Scale, the Spine-Check Score MediMouse® and per trunk rotation measurements. Prior to the start, directly after the intervention week and also during the observation period, chronicity, pain, physical and psychological impairment as well as disability due to cLBP (chronic low back pain) of all subjects were assessed via different validated questionnaires (Oswestry Low Back Disability Index, Medical Outcomes Study Short Form 36, modified Visual Analogue Scale, World Health Organization Well-Being Index). In a pain diary, the use of pain medication was documented during the whole study period. Furthermore, the days of incapacity to work and the number of medical consultations due to cLBP in the last months were assessed. These three parameters were collected two times (day 0 and day 120).

#### Back performance scale (BPS)

The BPS is an assessment of mobility-related activities in patients with back pain. It is seen as a reliable and valid instrument for measuring functional spinal mobility and to detect relevant clinical changes [23, 24]. The BPS includes five movements, which are based on the activities of daily life (ADL): sock test, lifting test, pick-up test, roll-up test and finger-to-floor test [25]. In this study, only the first two movements (sock test on both sides and lifting test) were performed. The worst measured score during the assessment was recorded – the fewer points the patients reached, the better the functional mobility of their back (maximum possible value per test: 3 points).

#### Spine-check score©

The MediMouse® (Idiag, Switzerland) is a computer-supported skin surface device for measuring lumbar, thoracic and sacral spinal curvature and sagittal range of motion. The MediMouse® is guided along the spine of the patient from the spinous process of the 7th cervical vertebra to the spinous process of the 3rd sacral vertebra, recording the length and contour of the spine. The electronic 3D sensors simultaneously detect the local inclination in all three levels of the room. This information is transmitted via Bluetooth to the PC and evaluated by the software. By intersegmental angles this test calculates the Spine-Check Score© which includes the three criteria of posture, mobility and postural stability. The examination is carried out in the upright position, in flexion and a loaded recording of the spine column (Matthias test) [26]. Since the validity and quality of the recording depends strongly on the applied pressure of the instrument, the instruction and variations of body positioning [27], the MediMouse® test was carried out a total of three times and the mean value of these three measurements was used for further calculation. The overall outcome of the Spine-Check Score© was calculated according to the following weighting: mobility 40%, postural stability 40% and posture 20%. The results were interpreted as follows: the lower the score, the more abnormalities exist. With great range of movement, values of degrees were higher.

#### Measurement of torso rotation

For the elevation of the individual trunk rotation degree, the patients were sitting on a treatment bed, while their feet had ground contact. A bar was placed over the shoulders behind the cervical spine and fixed with both hands. The patients were then asked to turn the trunk as far as possible to one side (right or left). It was important that the execution took place without pain and that the motion was not carried out abruptly. The mobility was measured with a digital goniometer (Stabilized Compass AndroidApp, Anagog Software, Tel Aviv, ISR). This survey was repeated three times for each side, the mean value was used for further calculation.

#### Oswestry low Back disability index (ODI)

The ODI contains ten items related to pain behavior and daily activities of living that may be affected by cLBP [28]: pain intensity, personal care (e.g. washing or dressing), lifting, walking, sitting, standing, sleeping, social life, traveling and changing degree of pain. The lower points achieved, the lower the impairment in everyday life. A combination of high reliability (on intra-class consistency correlation and on the test-retest) and validity (especially on construct validity) was found in the ODI and it appeared to react sensitive with meaningful clinical changes [29–31].

#### Modified visual analogue scale (mVAS)

A modified visual analogue scale for pain and status of health assessed the subjective pain intensity of the patients. The Visual Analogue Scale is a unidimensional measuring instrument, often used for the pain monitoring of adults [32]. The patients were asked to report their “current” pain intensity and subjective status of health - on one scale each. On the front of these scales there is a verbal question (e.g. “How do you judge your current health status?”), as well as a comic strip with six faces that provides an orientation for the intermediate state of the two parameters. A revised faces pain scale supplemented the mVAS. On the back there is a scale of 0 to 100 mm to quantify the momentary situation of the patients in a valid and reliable way, whereby a higher value indicates a better clinical result (lower pain intensity as well as a better health status) [33–35].

#### Medical outcomes study short form 36 (SF-36)

The SF-36 is a health status scale for measuring the subjective health-related quality of life. It includes the following eight dimensions of health: physical function, role behavior due to physical impairment, pain, general health, vitality and physical energy, social functioning, role behavior due to mental impairment, as well as mental function [36]. The SF-36 questionnaire includes some of the most frequently measured health concepts and is used as a valid health measure for documenting the burden of a disease [37, 38].

#### World Health Organization well-being index (WHO-5)

The WHO-5 is a brief self-contained questionnaire including five positively formulated phrases, which refer to positive mood, vitality and general interests for screening depression in chronic illness: good spirits, relaxation, being active, waking up fresh and rested, and being interested in things are topics of it [39]. The answers of this test result in a sum value with a low total value corresponding to a low level of well-being. WHO-5 Index is seen as a sensitive, specific [40] and effective assessment [39], which is applied successfully in various study fields [41].

### Statistical analysis

Statistical analyses were performed using the SPSS software (IBM Corp. Released 2016. IBM SPSS Statistics for Windows, Version 24.0. Armonk, NY: IBM Corp). Statistical significance was set at the 0.05 probability level for all tests and is expressed as *p* ≤ 0.05 (*), as *p* ≤ 0.01 (**) or as *p* ≤ 0.001 (***). For the per protocol analysis, missing values were not replaced. Evaluation of the data distribution was performed by Kolmogorov-Smirnov (with Lilliefors correction of significance) and Shapiro-Wilk normality test and yielded that more than 60% of the generated data follows normal distribution. Due to the smaller sample sizes in the three subgroups, the Shapiro-Wilk test was added for the individual evaluation of normal distribution in the GEBT, GE and CO group [42]. To identify significant differences between the groups at baselines, ANOVA was performed for parametric data and Kruskal-Wallis for non-parametric data, both including Bonferroni correction.

Linear mixed models (LMM) with treatment and time or treatment, time and the interaction of treatment and time as fixed factors were used to analyze the effects of the treatments over time. To account for individual differences, the patient ID was set as random effect for all models. In LMM1 the treatments GEBT and GE vs. CO, time and the interaction of treatment and time were included. In LMM2 the treatments GE vs GBT, time and the interaction of treatment and time were evaluated. In LMM3, only treatment (GEBT and GE vs. CO) and time were included.

For measurements concerning claim of medical care (pain medication, physicians’ consultations, status of employee’s illness), taken at two instants of time, Friedman test (non-parametric data) was applied. Means and corresponding standard deviations can be found in Table 6 in Appendix. Baseline characteristics expressed as mean ± SD are shown in Table 5 in Appendix.

## Results

### Demographics and patients’ characteristics

This randomized controlled clinical study comprised 80 persons with diagnosed nscLBP consisting of 35 men and 45 women (for full demographics see Table 5 in Appendix).

The CO group has a bias in age and is slightly younger compared to the GEBT and GE group. With the exception of the parameters physician’s consultations, employee’s illness, Spine-Check Score© postural stability, spine rotation on both sides and mVAS pain, there are no statistically significant baseline differences between the three treatment groups (Table 2, Table 5 in Appendix).

**Table 2:** Baseline characteristics of study participants (full version see Table 5 in Appendix)

	Green exercise and balneotherapy	Green exercise	Control	*p*-value with Bonferroni correction	
Number	26	27	27		
Sex (female/male)	14/12	14/13	17/10		
Age (years)	53.35 ± 8.26	52.85 ± 6.43	43.81 ± 12.07	< 0.000***	
BMI	26.32 ± 4.47	24.78 ± 2.73	25.06 ± 3.18	0.245	
Korff	1.92 ± 1.02	1.52 ± 0.75	1.63 ± 0.77	0.580	
Pain medication	0.73 ± 1.04	0.74 ± 1.1	1 ± 0.89	0.368	
Physicians consultations	0.88 ± 1.28	0.63 ± 0.79	1.37 ± 0.63	0.002**	
Status of employee’s illness	1 ± 1.67	0.3 ± 0.87	0.89 ± 0.64	0.002**	
Job satisfaction	74.38 ± 25.21	79.19 ± 20.59	79.96 ± 23.16	1	

Data represented as mean ± SD

**Fig. 2:**
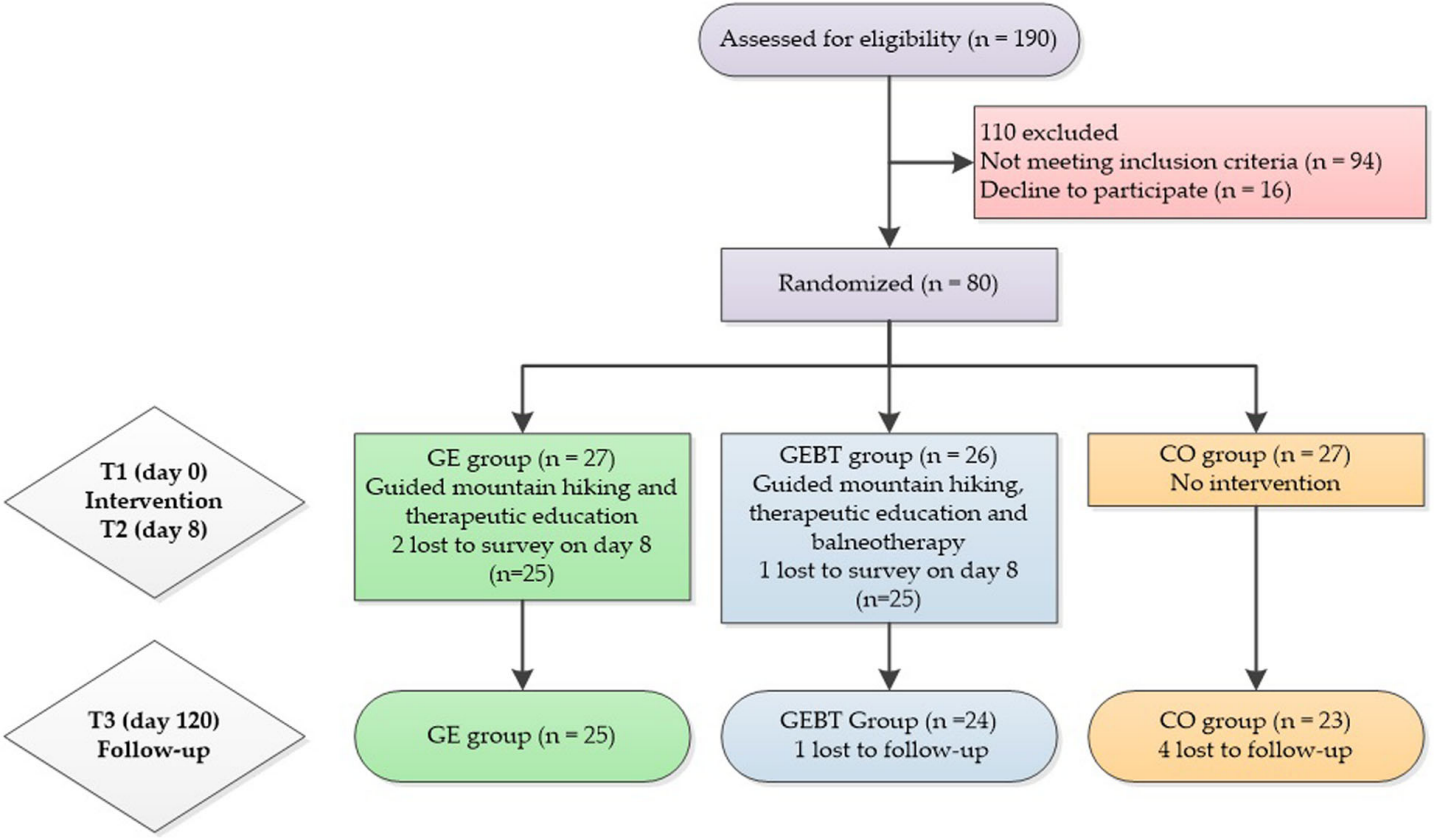
Patient Recruitment. Numbers of included and excluded patients

Patients could cancel their participation in this clinical trial at any time without giving reasons. (Fig. 2). All dropouts were voluntarily decided by the participants. There was no injury during the intervention and follow-up period.

### Functional spine mobility

**Fig. 3:**
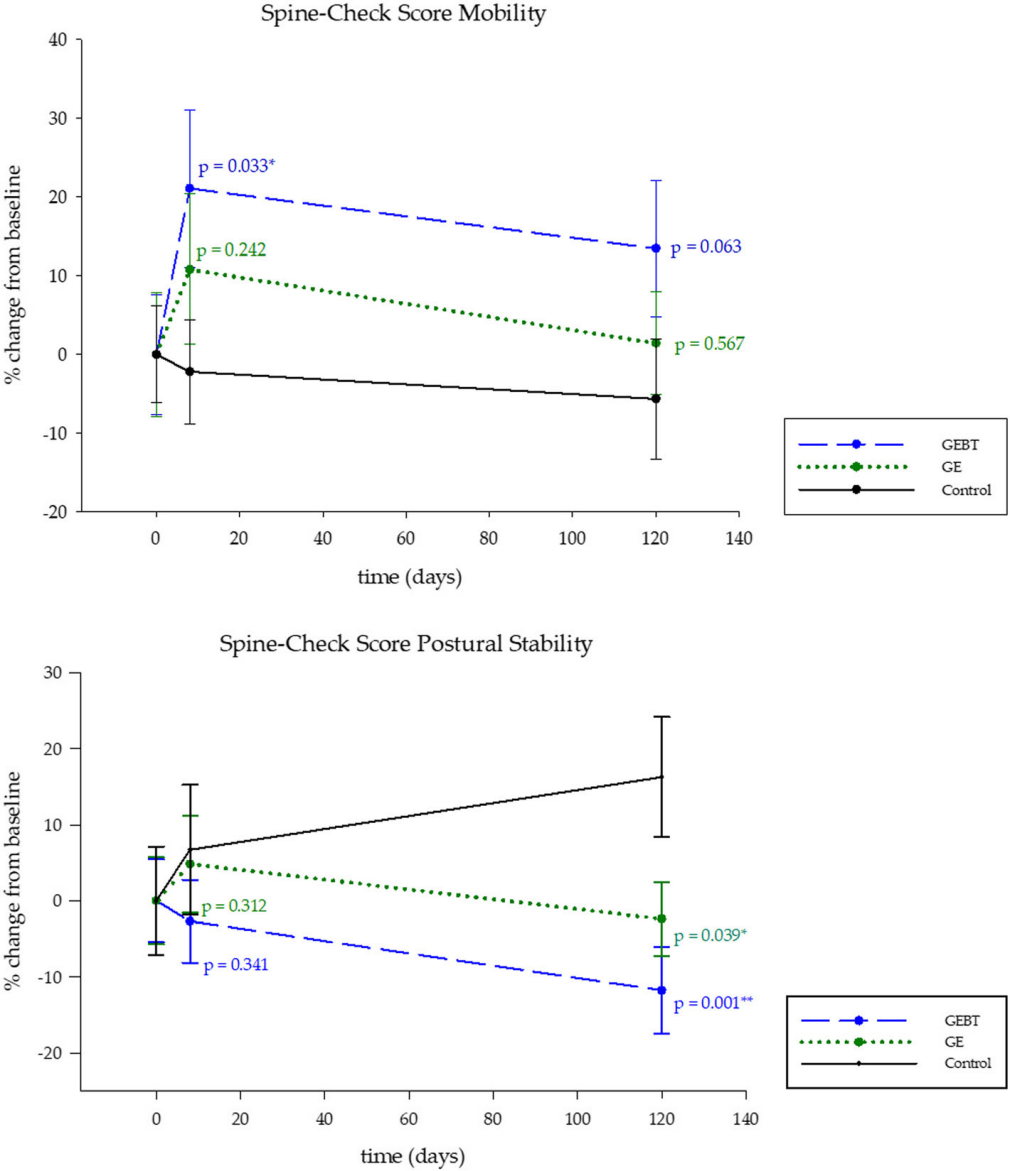
Spine-Check Score Mobility and Postural Stability. Functional spine mobility. Mobility and postural stability of the Spine-Check Score©. Linear mixed model of both intervention groups compared to the control group (LMM1). Data shown in percentage change from baseline (±SD), significances are indicated by asterisks. Means (±SD) of data are shown in Table 3

With respect to the criteria mobility, LMM1 analysis of the Spine-Check Score© displays a significant increase of values in the GEBT group (*p* = 0.033) on day 8, reflecting a higher mobility (Fig. 3, Table 3). Furthermore, LMM3 analysis shows a significant difference over time in both intervention groups on day 8 (*p* = 0.044) (Table 3).

**Table 3:** Linear mixed models

	GEBT and GE vs. Control						GEBT vs. GE		
	LMM1			LMM3			LMM2		
	fe	*p* value		fe	*p* value		fe	*p* value	
SCS© total									
	GEBT x T2	0.312	n.s.	GEBT	0.237	n.s.	GEBT x T2	0.837	n.s.
	GEBT x T3	0.362	n.s.	GE	0.085	n.s.	GEBT x T3	0.923	n.s.
	GE x T2	0.222	n.s.	T2	0.035	*			
	GE x T3	0.409	n.s.	T3	0.584	n.s.			
SCS© mobility									
	GEBT x T2	0.033	*	GEBT	0.694	n.s.	GEBT x T2	0.315	n.s.
	GEBT x T3	0.063	n.s.	GE	0.598	n.s.	GEBT x T3	0.167	n.s.
	GE x T2	0.242	n.s.	T2	0.044	*			
	GE x T3	0.567	n.s.	T3	0.582	n.s.			
SCS© postural stability									
	GEBT x T2	0.341	n.s.	GEBT	0.040	*	GEBT x T2	0.308	n.s.
	GEBT x T3	0.001	***	GE	0.007	**	GEBT x T3	0.200	n.s.
	GE x T2	0.967	n.s.	T2	0.325	n.s.			
	GE x T3	0.039	*	T3	0.940	n.s.			
SCS© posture									
	GEBT x T2	0.198	n.s.	GEBT	0.968	n.s.	GEBT x T2	0.581	n.s.
	GEBT x T3	0.910	n.s.	GE	0.547	n.s.	GEBT x T3	0.822	n.s.
	GE x T2	0.059	n.s.	T2	0.123	n.s.			
	GE x T3	0.900	n.s.	T3	0.410	n.s.			
BPS sock test right									
	GEBT x T2	0.870	n.s.	GEBT	0.224	n.s.	GEBT x T2	0.261	n.s.
	GEBT x T3	0.635	n.s.	GE	0.976	n.s.	GEBT x T3	0.981	n.s.
	GE x T2	0.305	n.s.	T2	0.307	n.s.			
	GE x T3	0.614	n.s.	T3	0.309	n.s.			
BPS sock test left									
	GEBT x T2	0.056	n.s.	GEBT	0.198	n.s.	GEBT x T2	0.101	n.s.
	GEBT x T3	0.534	n.s.	GE	0.859	n.s.	GEBT x T3	0.366	n.s.
	GE x T2	0.775	n.s.	T2	0.987	n.s.			
	GE x T3	0.797	n.s.	T3	0.732	n.s.			
BPS lifting test									
	GEBT x T2	0.584	n.s.	GEBT	0.183	n.s.	GEBT x T2	0.882	n.s.
	GEBT x T3	0.299	n.s.	GE	0.256	n.s.	GEBT x T3	0.410	n.s.
	GE x T2	0.675	n.s.	T2	0.000	***			
	GE x T3	0.751	n.s.	T3	0.000	***			
Spine rotation right									
	GEBT x T2	0.072	n.s.	GEBT	0.000	***	GEBT x T2	0.796	n.s.
	GEBT x T3	0.321	n.s.	GE	0.000	***	GEBT x T3	0.995	n.s.
	GE x T2	0.121	n.s.	T2	0.001	***			
	GE x T3	0.328	n.s.	T3	0.410	n.s.			
Spine rotation left									
	GEBT x T2	0.597	n.s.	GEBT	0.157	n.s.	GEBT x T2	0.535	n.s.
	GEBT x T3	0.871	n.s.	GE	0.028	**	GEBT x T3	0.917	n.s.
	GE x T2	0.231	n.s.	T2	0.662	n.s.			
	GE x T3	0.785	n.s.	T3	0.088	n.s.			
mVAS pain									
	GEBT x T2	0.135	n.s.	GEBT	0.249	n.s.	GEBT x T2	0.903	n.s.
	GEBT x T3	0.002	**	GE	0.589	n.s.	GEBT x T3	0.308	n.s.
	GE x T2	0.105	n.s.	T2	0.048	*			
	GE x T3	0.035	*	T3	0.166	n.s.			
mVAS health status									
	GEBT x T2	0.026	*	GEBT	0.611	n.s.	GEBT x T2	0.755	n.s.
	GEBT x T3	0.015	*	GE	0.394	n.s.	GEBT x T3	0.190	n.s.
	GE x T2	0.054	n.s.	T2	0.000	***			
	GE x T3	0.258	n.s.	T3	0.000	***			
mVAS health behaviour									
	GEBT x T2	0.881	n.s.	GEBT	0.730	n.s.	GEBT x T2	0.222	n.s.
	GEBT x T3	0.074	n.s.	GE	0.168	n.s.	GEBT x T3	0.037	*
	GE x T2	0.187	n.s.	T2	0.184	n.s.			
	GE x T3	0.815	n.s.	T3	0.065	n.s.			
WHO-5									
	GEBT x T2	0.001	***	GEBT	0.844	n.s.	GE x T2	0,191	n.s.
	GEBT x T3	0.229	n.s.	GE	0.125	n.s.	GE x T3	0,247	n.s.
	GE x T2	0.057	n.s	T2	0.000	***			
	GE x T3	0.999	n.s.	T3	0.150	n.s.			
ODI									
	GEBT x T2	0.103	n.s.	GEBT	0.837	n.s.	GEBT x T2	0.779	n.s.
	GEBT x T3	0.266	n.s.	GE	0.234	n.s.	GEBT x T3	0.598	n.s.
	GE x T2	0.169	n.s.	T2	0.000	***			
	GE x T3	0.551	n.s.	T3	0.000	***			
SF36 total									
	GEBT x T2	0.026	*	GEBT	0.978	n.s.	GEBT x T2	0.539	n.s.
	GEBT x T3	0.071	n.s.	GE	0.669	n.s.	GEBT x T3	0.229	n.s.
	GE x T2	0.108	n.s.	T2	0.002	**			
	GE x T3	0.557	n.s.	T3	0.000	***			
SF36 physical health									
	GEBT x T2	0.023	*	GEBT	0.874	n.s.	GEBT x T2	0.449	n.s.
	GEBT x T3	0.058	n.s.	GE	0.439	n.s.	GEBT x T3	0.794	n.s.
	GE x T2	0.106	n.s.	T2	0.000	***			
	GE x T3	0.104	n.s.	T3	0.000	***			
SF36 mental health									
	GEBT x T2	0.104	n.s.	GEBT	0.663	n.s.	GEBT x T2	0.829	n.s.
	GEBT x T3	0.168	n.s.	GE	0.723	n.s.	GEBT x T3	0.058	n.s.
	GE x T2	0.161	n.s.	T2	0.009	**			
	GE x T3	0.550	n.s.	T3	0.040	*			

*P*-values of three linear mixed models (LMM). LMM1: GEBT and GE vs. CO, time and interaction of treatment and time; LMM2: GEBT vs. GE, time and interaction of treatment and time; LMM3: GEBT and GE vs. CO, time; Day 8 (T2) and day 120 (T3). Level of significance ≤0.05 *, level of significance ≤0.01 **, level of significance ≤0.001 ***. Fe (Fixed effect), SCS© (Spine-Check Score©), BPS Back performance scale, SCS© (Spine-Check Score©), mVAS (modified Visual Analogue Scale), WHO-5 (World Health Organization Well-Being Index), ODI (Oswestry Disability Index), SF-36 (Medical Outcomes Study Short Form 36)

Regarding the postural stability, LMM1 analysis observes significantly lower values in both intervention groups on day 120 (GEBT: *p* = 0.001; GE: *p* = 0.039) (Fig. 3). LMM3 analysis of the total value of the Spine-Check Score© detects changes over time on day 8 (*p* = 0.035) (Table 3). Referring to the remaining measurements of the Spine-Check Score©, no significant differences between the groups could be detected.

No significant differences between the groups concerning to the items of the Back Performance Scale could be detected via LMM1, but the model excluding the interaction of time and treatment (LMM3) shows significant changes over time in both intervention groups at all time points (day 8 *p* <  0.000, day 120 *p* <  0.000) for the lifting test (Table 3).

LMM3 analysis of the trunk rotation to the right side detects significant changes over time in both intervention groups on day 8 (p = 0.001). Looking at the total trunk rotation, both intervention groups show a significant change in treatment (GEBT *p* = 0,001; GE *p* = 0,000).

### Pain behavior and daily activities of living

**Fig. 4:**
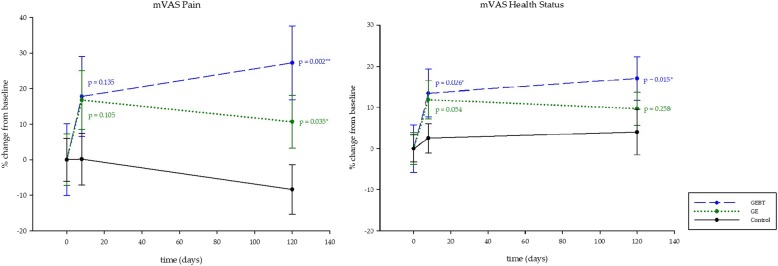
Pain and Health Status. Pain behavior and health status of the patients assessed with the mVAS. Statistical analysis calculated with Linear mixed model (LMM1). Asterisks indicate significances. Data shown in percentage change from baseline (±SD). Data means (±SD) are shown in Table 3

Results of the mVAS indicate a clear long-term trend of a beneficial effect in the GEBT group concerning pain intensity, exhibited in a significant pain relief on day 120 (LMM1, *p* = 0.002). Also, the GE group showed a long-term effect on day 120 (LMM1, *p* = 0.035) (Fig. 4, Table 3). It is essential to mention that the patients in the GEBT group evince higher pain intensity in the baseline measurement than those in the GE and CO group, as shown in Table 3. LMM3 analysis of the mVAS pain scale detects a change over time on day 8 (*p* = 0.048) (Table 3).

LMM1 analysis of the values from the mVAS health status scale demonstrate significant improvements of the GEBT group: day 8 *p* = 0.026, day 120 *p* = 0.015. Analysis via LMM3 also reports positive significant changes in both intervention groups (day 8: *p* <  0.000; day 120: *p* <  0.000) (Table 3).

Regarding the health behavior assessed with the mVAS, there is a significant difference in favor of the GEBT group on day 120 (LMM2, *p* = 0.037) (Table 3).

The Oswestry Disability Index (ODI), a measurement for pain behavior and activities of daily living for patients with cLBP, recorded significant changes in both intervention groups over time at all measurement points (LMM3; day 8: *p* <  0.000, day 120, p <  0.000). Both the ODI and the Korff graduation scale, monitoring pain related disability, elicit trends for improvement in all study arms (LMM 1), but no significant differences over time between the three study groups could be observed (Table 3).

### Subjective health-related quality of life

**Fig. 5:**
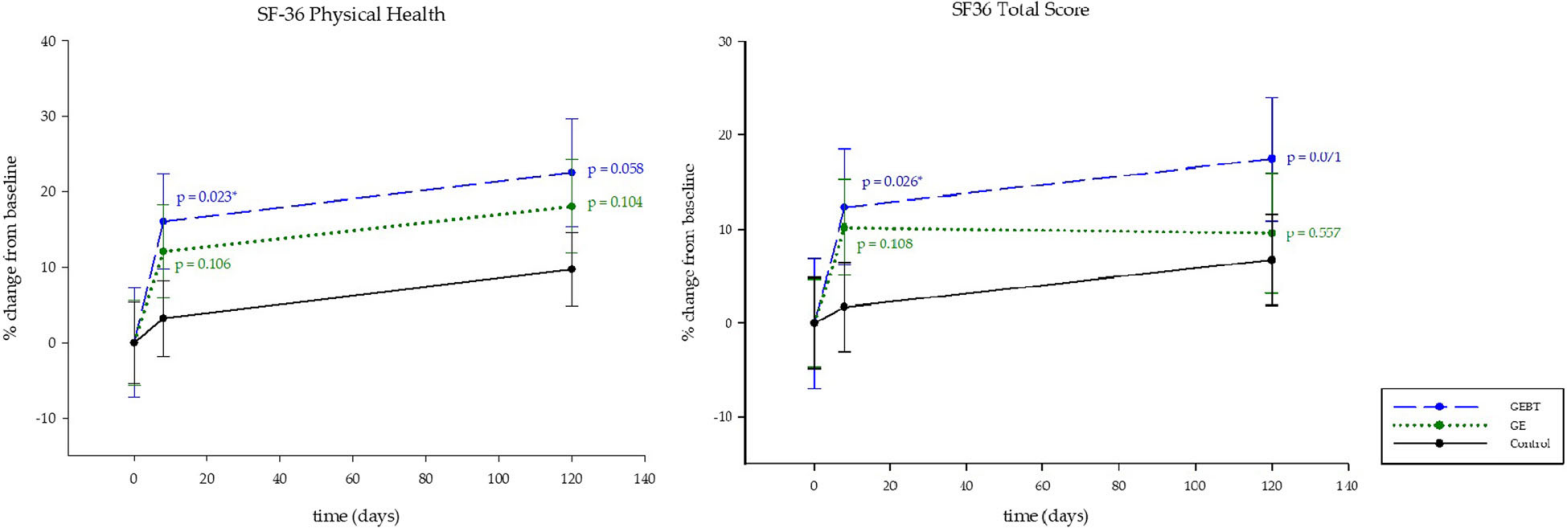
Health-related Quality of Life. Subjective health-related quality of life. The category physical health and the total score of Medical Outcomes Study Short Form 36 (SF-36) shown in percentage change from baseline (±SD). The linear mixed model (LMM1) of three study groups over time indicates significant beneficial effects in the GEBT group. Asterisks indicate significances; data means (±SD) are shown in Table 3

For the GEBT group, LMM1 analysis of the SF-36 reveals improvement in two of the three test categories of the SF-36 questionnaire, on day 8 (physical health: *p* = 0.023; total score: p = 0.026). The GE group showed no significant changes (Fig. 5). The linear model without interplay of time and treatment (LMM3) shows significant alteration over time on day 8 (*p* <  0.000) as well as on day 120 (*p* <  0.000) in both intervention groups concerning physical health (Table 3). Regarding the SF-36 total score, LMM3 analysis reports significant changes in both intervention groups (day 8: *p* = 0.002; day 120: *p* <  0.000) (Table 3).

Concerning the category mental health of the SF-36 questionnaire, LMM3 analysis recorded significant changes over time in the GE and GEBT group on day 8 (*p* = 0.009) and day 120 (*p* = 0.040) (Table 3).

### Depression in chronic illness

**Fig. 6:**
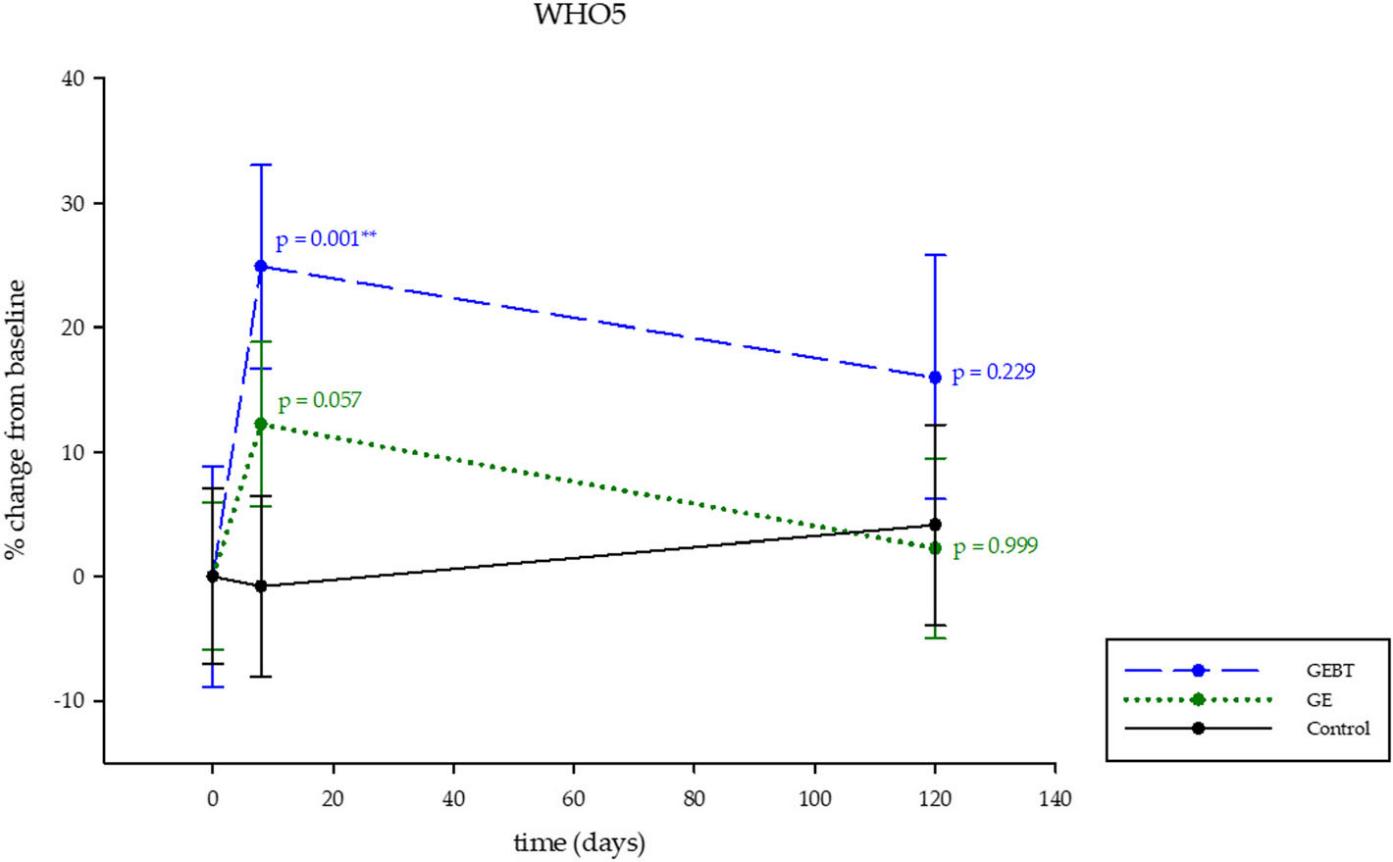
Depression in Chronic Illness. Depression in chronic illness, the WHO-5 questionnaire. Significantly decreased depression levels in the GEBT group on day 8 (LMM1), indicating a specific, balneotherapeutic effect. Data shown in percentage change from baseline (±SD), means (±SD) of data are given in Table 3. Significances are indicated by asterisks

A significant higher well-being level could be measured by LMM1 analysis in the GEBT group after the intervention week on day 8 (*p* = 0.001) for the WHO-5 Index (Fig. 6, Table 3). LMM3 analysis demonstrates significant changes over time on day 8 (*p* <  0.000) in both intervention groups (Table 3).

### Claim of medical care

All three study groups show an improvement over time (Friedmann test; pain medication *p* <  0.000; physicians consultations *p* <  0.000; status of employee’s illness *p* <  0.000), but no statistically valid differences in-between the groups on day 120 (Kruskal-Wallis test) (Table 6 in Appendix).

## Discussion

The present controlled and randomized clinical trial addressed the question, whether green exercise (moderate mountain hiking) alone or in combination with Mg-Ca-SO_4_ thermal balneotherapy provides beneficial effects for the treatment of non-specific chronic low back pain.

As indicated by our results, it can be assumed that green exercise has the greatest therapeutic influence, besides the social component of the group constellation as an important contributing factor. Commonly used in multimodal treatments of the rehabilitative care sector, the impact of positive group dynamics is a well-known influencing aspect [6]. This assumption is justified by the fact, that hardly any significant differences could be found in statistical analysis of our data by means of the two-armed linear mixed models (LMM2, GEBT group compared to GE group), but many signifiers in comparison to the control group (LMM1, LMM3).

Most data feature a high standard deviation. This could be attributed to the fact, that stratification by the Korff assessment achieved an optimal distribution of the patients with regard to pain related disability, also reflected in subjective health-related quality of life, mobility and further assessments.

Since a pathophysiological change of pain perception occurs in the brains of chronic pain patients (so-called pain memory) [43], a persistent significant reduction of pain after 1 week is not realistic to assume. However, cortical plasticity, which is important in chronic pain conditions, offers potential rehabilitation goals to be achieved by motoric, cognitive behavioral and sensory strategies. In our study, a green exercise approach (moderate mountain hiking) was adopted and an additional sensory component (relaxation and pleasant temperature during spa therapy) was used in the GEBT group. Regarding pain relief and health behavior after 1 week of intervention, the results in the GEBT group are particularly to be emphasized. Additionally, the surveys of subjective health-related quality of life as well as depression in chronic illness are indicators of the multidimensionality of the clinical pattern of nscLBP and showed a corresponding reaction to both interventions, but especially in the GEBT group.

Interestingly, the results of the functional spinal mobility are partly contradictory (e.g. day 8/ GEBT group: Spine-Check Score© mobility and trunk rotation total versus sock test right). A possible explanation for this could be avoidance mechanisms or movement patterns reflected in these tests, which chronic pain patients have appropriated over years (e.g. methods/tools to pull on socks).

Through different approaches like sight [44, 45], noise and smell during green exercise [45], natural environment has a direct and positive influence on physiological [46], as well as on psychological parameters [47, 48]. Unlike indoor exercises, physical outdoor activities offer opportunities which are associated with better health, like exposure to sunlight for adequate vitamin D levels [49] or a setting of a waterfall environment for beneficial, immunoregulatory effects [50]. In addition, there is a connection between green environments and the reduction of health inequality related to income deprivation [51], which may also have an impact considering the multiple factors influencing the genesis of nscLBP. Depending on certain therapy goals of care and cure, green exercise could be a useful and effective nature therapy or intervention program with health-supporting functions [52].

According to Rogerson et al., the social interaction time during outdoor exercise is significantly greater than during indoor sports [53]. Moreover, Gladwell et al. provide evidence for an increased participation in physical activities in natural environments, through enhanced enjoyment, a raised frequency and more social interaction [49]. However, the advantages of an indoor setting with regard to facilitated social interaction have been discussed by Hug et al. [54]. On the one hand, green exercise can afford occasions for social contacts, and on the other hand, it offers more opportunities for solitude and liberty from social pressures in comparison with indoor exercise settings.

The ideal characteristic of nature suitable for nscLBP-therapy is currently not investigated. In our study, significant positive effects of green exercise in patients suffering from nscLBP were obtained in a moderate alpine environment and can be recommend for this patient group. Since the average altitude at which the patients stayed during the hikes was a maximum of 1500 m above sea level, the influence of the altitude for the parameters collected in this study may be negligibly small. It would be interesting to investigate the influence of mountain hiking at high altitude (> 2000 m) on patients with nscLBP. In this regard, further physiological changes could be expected [55, 56].

In comparison to mountain hiking alone, the combination of green exercise and relaxation due to spa therapy has additional and sustainable therapeutic effects. This might be attributed to the thermal effect, which has an influence on patients via four pathways: vasodilatation, gate control mechanism, elevation of beta-endorphin levels and muscle relaxation [57]. The thermal effects could not only have reduced the patients’ pain intensity, but also shortened the time of super-compensation and regeneration after mountain hiking. Furthermore, immersion in the thermal water allowed the patients to mobilize the lower spine and its adjacent joints with minimal discomfort. The higher the mineral concentration of the water, the higher the hydrostatic pressure and the impact on the patient’s body. The total mineral concentration of the Grins water is ~ 2.2 g/l and therefore it can be classified as mildly mineralized [58]. We are in line with results of Gáti et al. [59], indicating balneotherapy in calcium-magnesium-sodium-bicarbonate might have a favorable impact on the clinical parameters of patients suffering from chronic low back pain. According the scientific literature on this subject, the terms “spa therapy” and “balneotherapy” are used for this type of intervention [60, 61]. There is also a strong placebo effect assigned to spa treatments, with the psychologic impact of removal being seen as an important aspect. The mechanism of experienced greater physical and mental quality of life and less depression may be adaptive modification in regulatory systems, especially of autonomous functions as well as behavioral changes [60].

As the development of an exercise program with biomechanical and aerobic features or a combination of approaches is recommended, the therapeutic approach of this study offers optimal implementation. The efficacy of this multimodal therapy approach is evident in its results and corresponds to current therapeutic recommendations, like the NICE guideline for management of low back pain [62].

## Limitations

NscLBP has a lot of influencing factors in its pathogenesis, as well as subgroups of pathology and heterogeneity in appearance [6, 8], which have not been considered in this study.

We did not record hiking with a small backpack, as most patients did in our study, as well as previous hiking experiences in this sport, which could affect the functional parameters especially.

Whether movement during the orthopedic tests was stopped by pain, by a structurally caused limitation, or by a non-trust in one’s own body ability was not recorded. We instructed to carry out all movements during examinations without pain or rather without an increase of continuously present pain.

The study did not address the lifestyle and activity level (continuation of hiking, exercise plan at home) of the patients within the follow-up time, which should be taken into account as possible important influencing factors regarding the results.

Additionally, regarding the intervention groups, social influences of common activity in a group must be mentioned, which might influence the results.

The absorption of minerals dissolved in Mg-Ca-SO_4_ thermal water was not investigated in this study and could be content of further research, because mineral concentration dependent effects may be linked to simple thermal effects [63]. However, especially mineral water, according to Morer et al. [64], has better and longer improvements in pain, function, quality of life as well as clinical parameters in some musculoskeletal diseases - including chronic low back pain - compared to baseline or non-mineral water treatments. For the comparison of Mg-Ca-SO_4_ thermal and tap water, a placebo balneotherapy could not have been used in this investigation because of the intense odor of Mg-Ca-SO4 water.

## Conclusions

Moderate mountain hiking and Mg-Ca-SO_4_ thermal spa therapy as a multimodal treatment of patients with nscLBP showed a benefit concerning pain, functional spine mobility, subjective health-related quality of life and depression in chronic illness. Balneotherapy seems to promote regeneration between physical demands and supports super-compensatory processes after mountain hiking tours. Based on our results, green exercise in moderate alpine environments and balneotherapy can be recommended as a fast acting and inexpensive therapy, which is easy to implement.

Because the intervention time of this study was 1 week, further studies are necessary, to examine long-term effects and the sustainability of this nature therapy.

## Appendix

**Table 4:** Water analysis Albenbad

	Thermal water^a^	Tab water	Unit	Test procedure
Electric conductivity	2317.5 ± 5	279	μS/cm	ÖNORM EN 2788
pH-Value	7.65 ± 0.15	8.02	(−log H+)	ÖNORM EN ISO 10523
Acid capacity	2.45 ± 0.01	2.88	mmol/l	ÖNORM EN 9963–1
Calcium	284 ± 9.09	34.4	mg/l	ÖNORM EN ISO 1185
Magnesium	234.75 ± 7.09	19	mg/l	ÖNORM EN ISO 1185
Potassium	1.60 ± 0.08	< 1	mg/l	ÖNORM EN ISO 1185
Sodium	3.8575 ± 0.23	< 1	mg/l	ÖNORM EN ISO 1185
Total hardness	94 ± 2.94	9.2	°KH	ÖNORM EN ISO 1185
Carbonate hardness	6.7	7.9	°KH	DIN 38409–6
Iron	< 0.01 (1 sample 0.07)	< 0.01	μg/l	ÖNORM EN 9963–1
Manganese	16.2 ± 4.23	8.88	μg/l	ÖNORM EN ISO 1185
Antimony	2.65 ± 0.58	16.7	μg/l	ÖNORM EN ISO 1185
Arsenic	26.15 ± 13.37	2.2	μg/l	ÖNORM EN ISO 1185
Hydrocarbonate	146.43 ± 0.53	172.7	mg/l	ÖNORM EN 9963–1
Chloride	1.17 ± 0.033	< 1	mg/l	ÖNORM EN ISO 10304-1
Fluoride	1.1	0.23	mg/l	ÖNORM EN ISO 10304-1
Sulphate	1489.25 ± 37.62	20.2	mg/l	ÖNORM EN ISO 10304-1
Nitrate	< 1	< 1	mg/l	ÖNORM EN ISO 10304-1
Ammonium	0.0337 ± 0.01 (1 sample < 0.05)	0.015	mg/l	ÖNORM EN ISO 11732
Nitrite	< 0,01	0.023	mg/l	ÖNORM EN ISO 13395
Phosphate, ortho	0.0195	< 0.01	mg/l	ÖNORM EN ISO 15681-2
Total organic carbon	< 0,5	1.16	mg/l	ÖNORM EN 1484
	(1 sample 0.64)			

*DIN* National German standard published by the German Institute for Standardization, *ÖNORM EN ISO* National Austrian standard published by the Austrian Standards Institute, *< BG* Less than limit of quantification;

^a^Thermal water was taken in four places (1 sample spring fitting, 2 samples water course, 1 sample faucet Albenbad), mean of the four samples ± SD is shown; if a sample was below limit of quantification and therefore not calculable, this is listed

**Table 5:** Baseline characteristics of study participants (completed list)

	Green exercise and balneotherapy	Green exercise	Control	*p*-value with Bonferroni correction
Number	26	27	27	
Sex (female/male)	14/2	14/13	17/10	
Age (years)	53.35 ± 8.26	52.85 ± 6.43	43.81 ± 12.07	< 0.000***
Height (m)	1.72 ± 0.9	1.73 ± 0.8	1.72 ± 0.8	0.773
Weight (kg)	78.39 ± 16.47	74.65 ± 11.83	74.13 ± 13.01	0.481
BMI	26.32 ± 4.47	24.78 ± 2.73	25.06 ± 3.18	0.245
Abdominal girth (cm)	96 ± 13.18	92 ± 9.1	90.59 ± 11.69	0.213
Systolic blood pressure	136.92 ± 19.21	131.41 ± 17.43	126.70 ± 11.84	0.067
Diastolic blood pressure	81.23 ± 11	78.70 ± 8.91	78.41 ± 8.24	0.494
Pulse (bpm)	69.58 ± 8	69.30 ± 11.51	65.65 ± 9.46	0.277
%p02	97.04 ± 1.56	97.26 ± 1.38	97.70 ± 1.56	0.218
Kids (n)	1.65 ± 1.16	2 ± 1.27	1.42 ± 1.17	0.518
Korff	1.92 ± 1.02	1.52 ± 0.75	1.63 ± 0.77	0.580
Pain medication	0.73 ± 1.04	0.74 ± 1.1	1 ± 0.89	0.368
Physicians consultations	0.88 ± 1.28	0.63 ± 0.79	1.37 ± 0.63	0.002**
Status of employee’s illness	1 ± 1.67	0.3 ± 0.87	0.89 ± 0.64	0.002**
Job satisfaction	74.38 ± 25.21	79.19 ± 20.59	79.96 ± 23.16	1
BPS sock test right	0.92 ± 1.2	0.52 ± 0.98	0.63 ± 1.04	0.622
BPS sock test left	0.73 ± 1.04	0.63 ± 0.88	0.59 ± 1.01	1
BPS lift test	0.46 ± 0.76	0.54 ± 0.81	0.78 ± 0.89	0.526
Spine rotation right	55.19 ± 21.90	59.41 ± 16.68	34.67 ± 13.46	< 0.000***
Spine rotation left	53.12 ± 24.76	58.11 ± 19.54	45.52 ± 11.98	0.041*
Spine-Check Score© total	50.86 ± 9.86	52 ± 13.07	47.93 ± 15.22	0.398
Spine-Check Score© posture	45.47 ± 15.59	46.53 ± 17.93	47 ± 12.74	0.936
Spine-Check Score© mobility	40.01 ± 15.61	42.65 ± 17.44	47.25 ± 15.13	0.432
Spine-Check Score© postural stability	64.47 ± 17.90	64.12 ± 19.01	48.25 ± 18.11	0.003**
ODI	22.31 ± 10.29	18.96 ± 8.35	19.70 ± 8.07	1.314
SF-36 total	60.63 ± 21.22	66.61 ± 16.16	66.95 ± 16.39	0.511
SF-36 physical health	55.88 ± 20.72	61.24 ± 17.97	61.47 ± 19.95	0.548
SF-36 mental health	63.6 ± 22.07	69.86 ± 15.78	68.72 ± 17.76	1
mVAS pain	48.62 ± 25.09	59.59 ± 22.41	64.08 ± 18.97	0.046*
mVAS state of health	62.49 ± 18.38	69.54 ± 13.99	69.65 ± 11.36	0.226
mVAS health behaviour	64.09 ± 17.52	69.05 ± 14.28	64.76 ± 17.87	0.604
WHO-5	13.19 ± 6	16 ± 4.89	14.54 ± 5.23	0.410

Data are represented as the mean ± SD; *BMI* Body mass index, *BPS* Back performance scale, *ODI* Oswestry Low Back Disability Index, *mVAS* Modified Visual Analogue Scale, *SF-36* Medical Outcomes Study Short Form 36, *WHO-5* World Health Organization Well-Being Index, %p02 Oxygen partial pressure

**Table 6:** Mean values of all results (measurements on day 0, 8 and 120)

	Pain medication 0		Pain medication 120
GEBT	0.73 ± 1.04		0.50 ± 0.93
GE	0.74 ± 1.1		0.48 ± 0.87
Control	1 ± 0.89		0.25 ± 0.44
	Physicians consultations 0		Physicians consultations 120
GEBT	0.88 ± 1.28		0.50 ± 0.72
GE	0.63 ± 0.79		0.32 ± 0.56
Control	1.37 ± 0.63		0.42 ± 0.58
	Status of employee’s illness 0		Status of employee’s illness 120
GEBT	1 ± 1.67		0.22 ± 0.52
GE	0.3 ± 0.87		0.28 ± 0.84
Control	0.89 ± 0.64		0.13 ± 0.34
	SCS© total 0	SCS© total 8	SCS© total 120
GEBT	50.86 ± 9.86	54.41 ± 10.74	50.17 ± 9.58
GE	52 ± 13.07	56.47 ± 14.25	52.23 ± 9.9
Control	47.93 ± 15.22	48.38 ± 12.81	50.33 ± 11.45
	SCS© mobility 0	SCS© mobility 8	SCS© mobility 120
GEBT	40.01 ± 15.61	48.45 ± 20	45.39 ± 17.02
GE	42.65 ± 17.44	47.27 ± 20.36	43.27 ± 13.94
Control	47.25 ± 15.13	46.21 ± 16.18	44.58 ± 17.62
	SCS© postural stability 0	SCS© postural stability 8	SCS© postural stability 120
GEBT	64.47 ± 17.90	62.73 ± 17.55	56.89 ± 18.16
GE	64.12 ± 19.01	67.21 ± 20.51	62.59 ± 15.64
Control	48.96 ± 18.11	52.25 ± 21.75	56.93 ± 18.94
	SCS© posture 0	SCS© posture 8	SCS© posture 120
GEBT	45.47 ± 15.59	49.59 ± 16.23	46.31 ± 16.79
GE	46.53 ± 17.93	53.47 ± 17.37	49.33 ± 18.7
Control	47 ± 12.74	45.36 ± 12.91	49.1 ± 16.2
	BPS sock test right 0	BPS sock test right 8	BPS sock test right120
GEBT	0.92 ± 1.2	0.68 ± 1.15	0.83 ± 1.24
GE	0.52 ± 0.98	0.52 ± 1	0.44 ± 0.87
Control	0.63 ± 1.04	0.48 ± 0.89	0.46 ± 0.72
	BPS sock test left 0	BPS sock test left 8	BPS sock test left 120
GEBT	0.73 ± 1.04	0.92 ± 1.22	0.83 ± 1.05
GE	0.63 ± 0.88	0.52 ± 0.87	0.56 ± 0.92
Control	0.59 ± 1.01	0.44 ± 0.89	0.58 ± 0.83
	BPS lifting test 0	BPS lifting test 8	BPS lifting test 120
GEBT	0.46 ± 0.76	0.24 ± 0.44	0.25 ± 0.53
GE	0.54 ± 0.81	0.32 ± 0.63	0.17 ± 0.48
Control	0.78 ± 0.89	0.48 ± 0.75	0:38 ± 0.82
	Spine rotation right 0	Spine rotation right 8	Spine rotation right 120
GEBT	55.19 ± 21.90	58.96 ± 16.51	55.29 ± 20.6
GE	59.41 ± 16.68	63.32 ± 19.19	58.8 ± 16.06
Control	34.67 ± 13.46	46.07 ± 10.85	29.67 ± 11.04
	Spine rotation left 0	Spine rotation left 8	Spine rotation left 120
GEBT	53.12 ± 24.76	52.52 ± 23.23	57.92 ± 17.79
GE	58.11 ± 19.54	52.64 ± 14.7	62.04 ± 27.26
Control	45.52 ± 11.98	48.15 ± 20.92	50.75 ± 21.62
	mVAS pain 0	mVAS pain 8	mVAS pain 120
GEBT	48.62 ± 25.09	57.28 ± 27.31	61.88 ± 24.72
GE	59.59 ± 22.41	69.60 ± 24.54	65.96 ± 21.69
Control	64.08 ± 18.97	64.18 ± 21.70	58.71 ± 20.56
	mVAS health status 0	mVAS health status 8	mVAS health status 120
GEBT	62.49 ± 18.38	70.91 ± 18.13	73.13 ± 16.02
GE	69.54 ± 13.99	77.79 ± 16.11	76.27 ± 13.69
Control	69.65 ± 11.36	71.38 ± 11.57	72.42 ± 17.61
	mVAS health behaviour 0	mVAS health behaviour 8	mVAS health behaviour 120
GEBT	64.09 ± 17.52	64.67 ± 16.36	74.47 ± 12.81
GE	69.05 ± 14.28	74.99 ± 13.06	68.9 ± 13.98
Control	64.76 ± 17.87	65.5 ± 19.14	67.71 ± 15.4
	WHO5 0	WHO5 8	WHO5 120
GEBT	13.19 ± 5.98	16.48 ± 5.39	15.30 ± 6.2
GE	16 ± 4.9	17.96 ± 5.3	16.36 ± 5.79
Control	14.54 ± 5.22	14.42 ± 5.38	15.14 ± 5.51
	ODI 0	ODI 8	ODI 120
GEBT	22.31 ± 10.29	14.80 ± 9.92	14.09 ± 9
GE	18.96 ± 8.35	11.92 ± 8.13	12.75 ± 7.02
Control	19.70 ± 8.07	16.22 ± 8.44	14.96 ± 8.04
	SF36 total score 0	SF36 total score 8	SF36 total score 120
GEBT	60.63 ± 21.22	68.11 ± 18.67	71.2 ± 19.34
GE	66.61 ± 16.16	73.4 ± 16.87	73 ± 21.25
Control	66.95 ± 16.39	68.1 ± 15.9	71.45 ± 15.84
	SF36 physical health 0	SF36 physical health 8	SF36 physical health 120
GEBT	55.88 ± 20.72	64.86 ± 17.58	69.46 ± 19.11
GE	61.24 ± 14.97	68.66 ± 18.86	72.31 ± 19.09
Control	61.47 ± 16.95	63.42 ± 15.51	67.44 ± 17.7
	SF-36 Mental Health 0	SF-36 Mental Health 8	SF-36 Mental Health 120
GEBT	63.6 ± 22.07	69.15 ± 19.73	71.61 ± 19.79
GE	69.86 ± 15.78	76.25 ± 15.98	71.24 ± 23.55
Control	68.72 ± 17.76	70.12 ± 16.83	72.1 ± 17.64

Elevated data shown as mean ± SD. *SCS©* Spine-Check Score©, *BPS* Back performance scale, *SCS©* Spine-Check Score©, *mVAS* Modified Visual Analogue Scale, *WHO-5* World Health Organization Well-Being Index, *ODI* Oswestry Disability Index, *SF-36* Medical Outcomes Study Short Form 36

## Abbreviations

%p02: Oxygen partial pressure
< BG: Less than limit of quantification
A 1, 2 and 3: Appendix 1, 2 and 3
a. s. l.: Above sea level
ADL: Activities of daily life
ANOVA: Analysis of variance
BMI: Body mass index
BPS: Back Performance Scale
cLB: Chronic low back pain
CO group: Control group
DIN: National German standard published by the German Institute for Standardization
e.g.: For example, exempli gratia
fe: Fixed effects
GE group: Intervention group: Green exercise
GEBT group: Intervention group: Green exercise and balneotherapy
IBM: International Business Machines Corporation
ID: Identity
ISR: Israel
LBP: Low back pain
LMM 1, 2 and 3: Linear mixed models 1, 2 and 3
Mg-Ca-SO4 thermal water: Magnesium-calcium-sulfate thermal water
mVAS: Modified Visual Analogue Scale
n: Number of participants
nscLBP: Non-specific chronic low back pain
NY: New York
ODI: Oswestry Low Back Disability Index
ÖNORM EN ISO: National Austrian standard published by the Austrian Standards Institute
p: Probability value
PVA: Austrian federal pension fund, Pensionsversicherungsanstalt
RCT: Randomized controlled trial
SCS©: Spine-Check Score©
SD: Standard deviation
SE: Standard error
SF-36: Medical Outcomes Study Short Form 36
SPSS: Statistical Package for the Social Sciences
USA: United States of America
WHO-5: World Health Organization Well-Being Index
